# OGER++: hybrid multi-type entity recognition

**DOI:** 10.1186/s13321-018-0326-3

**Published:** 2019-01-21

**Authors:** Lenz Furrer, Anna Jancso, Nicola Colic, Fabio Rinaldi

**Affiliations:** 1 Institute of Computational Linguistics, University of Zurich, Andreasstr. 15, 8050 Zürich, Switzerland; 2 Fondazione Bruno Kessler, Via Sommarive, 18, 38123 Trento, Italy

**Keywords:** Named entity recognition, Concept recognition, Natural language processing, Machine learning

## Abstract

**Background:**

We present a text-mining tool for recognizing biomedical entities in scientific literature. OGER++ is a hybrid system for named entity recognition and concept recognition (linking), which combines a dictionary-based annotator with a corpus-based disambiguation component. The annotator uses an efficient look-up strategy combined with a normalization method for matching spelling variants. The disambiguation classifier is implemented as a feed-forward neural network which acts as a postfilter to the previous step.

**Results:**

We evaluated the system in terms of processing speed and annotation quality. In the speed benchmarks, the OGER++ web service processes 9.7 abstracts or 0.9 full-text documents per second. On the CRAFT corpus, we achieved 71.4% and 56.7% F1 for named entity recognition and concept recognition, respectively.

**Conclusions:**

Combining knowledge-based and data-driven components allows creating a system with competitive performance in biomedical text mining.

## Background

Text mining is often the only answer to retrieving specific information in the vast amount of biomedical scientific literature. Reliably extracting basic entities such as chemicals, genes/proteins, diseases, or organisms is the foundation of most approaches to text mining. The task of detecting spans of text that denote an entity of interest is usually referred to as *named entity recognition* (NER). It is commonly modeled as a tagging problem, where the text is a sequence of tokens that are classified as relevant or irrelevant and if multiple entity types are targeted assigned a type. In the closely related task of *concept recognition* (CR, often also referred to as *entity linking*, *normalisation*, or *grounding*), entities are additionally annotated with unique identifiers.

In terms of methodology, many approaches have been taken towards biomedical entity recognition. The evolution of methods reflects the advances that can be observed in all areas of natural language processing (NLP). Early systems were based on hand-written rules for extracting entities [1–3]. Over the last decade, supervised machine-learning systems have become very popular. For NER, Conditional Random Fields (CRF) have long dominated the field [4–7]. Knowledge-based approaches using hand-crafted resources like gazetteers are widespread among CR systems [8–11], even though data-driven components are used frequently to generate and/or rank entity candidates [12–16]. In multi-model architectures, multiple models are combined in a serial [17] or parallel manner (ensemble systems) [18–20] or use unlabeled data for improving domain representation [21]. Approaches to tackling both NER and CR include sequential pipelines [22] and joint models [23, 24]. Very recently, the renaissance of neural networks (NN) observable in many subfields of artificial intelligence and NLP finally found its way to NER for the biomedical domain [25–29] and even CR [30].

In this work, we present OGER++, a hybrid NER-CR system for text mining in the biomedical domain. It combines a fast, dictionary-based entity recognizer and normalizer with a corpus-based disambiguation filter. The technical and qualitative performance of previous versions were described in [31, 32], respectively. More recently, we wrote an application based on OGER for the OpenMinTeD platform [33]. In the present work, we describe new experimental results and benchmarks performed with the current version of the software. The code base of OGER is freely available from https://github.com/OntoGene/OGER; the demo web service is running at https://pub.cl.uzh.ch/purl/OGER.

## Methods

OGER—OntoGene’s Entity Recognizer—is a versatile, extensible software package written for multiple applications. It can be used as a Python library, executed as a command-line tool, or run as a REST server with an API and a browser interface. Through a software hook, it can be extended by the user through custom Python modules. By OGER++, we refer to a publicly accessible web service hosted at our institute which provides on-the-fly document annotation using a large terminology resource and corpus-based disambiguation. In the following, we describe this actively running instance of OGER.

OGER++ performs document annotation in four steps: (1) document structure parsing, (2) entity recognition/normalization, (3) disambiguation, (4) serialization. A wide range of input and output formats are supported (Steps 1 and 4), including plain-text, PubMed/PMC XML, BioC [34] (XML and JSON), and PubAnnotator JSON [35, 36], among others. In the format conversion, textual content and basic structure (sections) are retained, as well as a limited set of metadata (document ID for most formats, all metadata for BioC). In Step 2, a dictionary-based strategy is applied to locate mentions of biomedical entities in the text and link them to identifiers of curated terminology resources, as described in the next section. This procedure frequently generates ambiguous annotations (i.e. the same text span is linked to multiple entities), which are addressed in Step 3, as discussed in the subsequent section.

### Dictionary-based entity recognition and normalization

OGER has its roots in the OntoGene term annotation pipeline, a knowledge-based information extraction system for scientific biomedical literature that has been successfully applied to a range of entity types (genes/proteins, chemicals, diseases, among others [37–40]). It has been reimplemented from scratch in Python and is being developed continuously. As such, it has seen considerable improvements in terms of flexibility and processing speed.

The core recognition procedure relies on a list of target terms (the dictionary), which are connected to entity identifiers. Since extraction with an exact-match strategy would lead to very low coverage, we perform a series of preprocessing steps which have a normalizing effect. For example, the text is tokenized in an aggressive, lossy way which collapses spelling alternations like e.g. “SRC1”/“SRC 1”/“SRC-1” into a single representation. A more detailed description of the preprocessing steps can be found in [41].

**Fig. 1:**
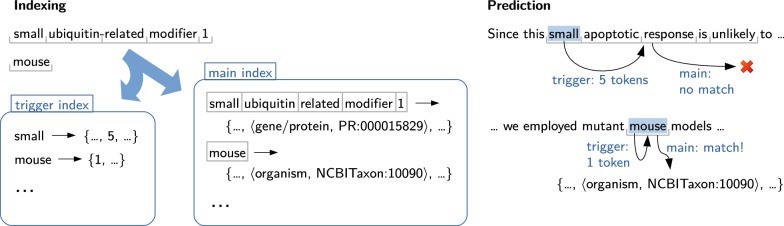
Term indexing using two hash tables. The examples illustrate how dictionary entries are indexed (left) and how the look-up is performed (right)

At indexing time, each term (name) from the dictionary is converted to a sequence of tokens through the same preprocessing steps that are used for the documents (see Fig. 1 for an example), thus assuring that all potential matchings will be preserved. These token sequences are indexed in a hash table, which maps the term to its dictionary entry (containing the identifier and other metadata). In case of ambiguity (multiple entries have the same token sequence), the value of the hash table will contain multiple entries; for synonyms (multiple terms for the same concept), multiple entries are indexed. For an efficient look-up of variable-length sequences, an additional hash table maps the first token of a term (trigger) to the length of the token sequence. At prediction time, each token of the text (preprocessed the same way as the dictionary terms) is looked up in the trigger index. If a match is encountered, candidate token sequences of appropriate length are extracted from the text, starting from the matching token. The extracted sequences are then looked up in the main index. Thanks to the trigger index, the number of look-ups per token is 1 in the common case (no trigger), i.e. complexity class *O*(*s*) (best case) with respect to the number of tokens per sentence. Using only the main index, a look-up would be required for each contiguous subsequence of the sentence, i.e. $O(s^2)$ or, if the token count of the longest entity is known, $O(s\times t_\text {max})$.

For the present work, we used two different configurations of terminology resources. In the experiment for evaluating annotation quality, we used the ontologies included in the CRAFT corpus [42], i.e. ChEBI [43], Cell Ontology [44], Gene Ontology [45], NCBI Taxonomy [46], Protein Ontology [47], and Sequence Ontology [48]. For the speed benchmarks, we used the default configuration of OGER’s web service, which uses up-to-date versions of the resources mentioned above and, in addition, Cellosaurus [49], CTD chemicals and diseases [50], MeSH [51], Swiss-Prot [52], and Uberon [53]. All resources were aggregated and converted to a unified format using the Bio Term Hub, a meta-resource for collecting and combining curated terminology resources [54].

### Corpus-based disambiguation

The dictionary-based concept-recognition module produces many spurious annotations. Words from the common vocabulary may be erroneously annotated as a biomedical entity (such as lead), and some terms are linked to identifiers of the wrong entity type (this often happens with abbreviations). Since OGER can produce multiple annotations for the same text span, the list of annotations might contain both correct and wrong results. Therefore, we augmented OGER with a postfilter component that removes spurious annotations.

**Fig. 2:**
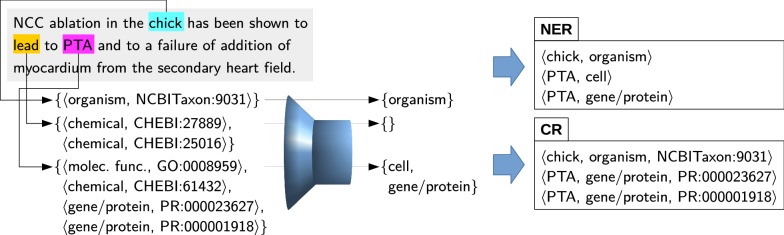
Example illustrating the disambiguation procedure. The corpus-based postfilter accepts, rejects, or reclassifies annotations from the upstream concept-recognition module

**Fig. 3:**
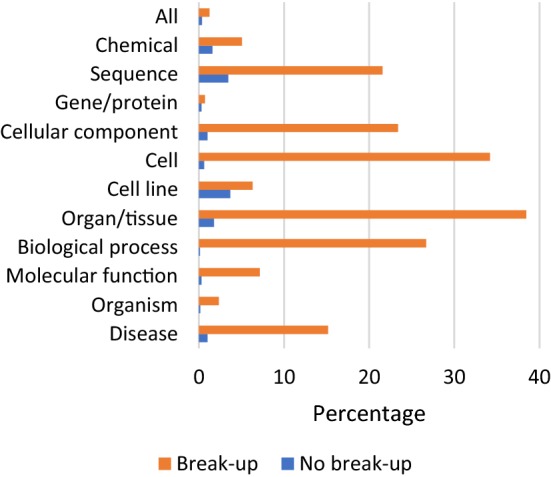
Percentage of terms occurring in Hunspell

The disambiguation procedure is illustrated in Fig. 2. For each annotated text span, the postfilter predicts a probability distribution over all entity types, including a label for not an entity. In the experiment with the CRAFT corpus (where a single text span can have multiple annotations), we applied the following heuristic to produce a label:

1. consider the highest-ranked entity type;
2. if the score difference between the two top-ranked types is less than a fixed threshold $\theta$, consider the second-ranked entity type as well;
3. remove occurrences of not an entity from the list of labels to be considered.

The threshold $\theta$ was empirically set to 0.3 based on hyperparameter optimization with 5-fold cross-validation on the training set. This heuristic produces zero, one, or two labels per text span, which are not necessarily a subset of the annotations originally generated by OGER. Depending on the task, they are used differently: In the case of NER, the produced labels are emitted directly. This means that an annotation might be re-classified, i.e. given an entity type that was not among OGER’s annotations. For the CR task, however, the concept identifiers are needed, therefore the original OGER annotations are used, restricted to the entries that match the postfilter’s output. This means that any re-classified annotation is lost in CR, since no identifier can be provided.

The postfilter module is a machine-learning-based classifier that has to be trained on an annotated corpus. In the present work, we used the CRAFT corpus [42], which is a collection of 67 full-text articles manually annotated for multiple entity types. The annotations cover chemicals, cell types, cellular components, organisms, genes/proteins, sequence features and the non-physical types biological processes and molecular functions. For our experiments, we excluded gene annotations linked to NCBI Gene (Entrez Gene) and conflated biological processes and molecular functions into a shared type BPMF. Annotations consisting of textually separated components were split into multiple, contiguous annotations. We divided the corpus into 47 documents for training and 20 for testing, using the same split as in our previous work [32].

The postfilter is implemented as a feed-forward neural network (NN). The initial design [32] was revised later [55] and integrated into OGER++. The key differences between the first and the current system are described in the following.

Firstly, both feature extraction and training of the NN is now performed in Python, thereby making it seamlessly work with the knowledge-based system implemented in the same programming language. The former system relied on a Java framework specialized on key-phrase extraction, plus a specialized learning module in R, to accomplish these tasks, thus making it very cumbersome to use in a pipeline. Secondly, a larger set of features was included as input to the NN. All thirteen features from the previous work were re-implemented. Four additional features were devised and evaluated:

- The **vowel:consonant** feature computes the proportion of vowels and consonants. Lower vowel counts are typical for certain entity types such as proteins.
- The **common vocabulary** feature computes whether the n-gram occurs in a common-language dictionary such as Hunspell [56]. Biomedical entities are less likely to appear in a common dictionary as can be seen in Fig. 3. Thus, this feature can help in deciding whether an n-gram should be ruled out as a biomedical entity mention. As Hunspell is intended to be used on single words, the percentages of terms known to Hunspell were calculated in two ways: In the “break-up” setting, the words of a term are looked up individually, while in the “no break-up” setting, they are passed to Hunspell as a whole. In the latter case, Hunspell always returns multi-word terms as not occurring in the dictionary. For some entity types, there are marked differences in the two percentages, notably for cells, biological processes, cellular components, sequences and organ/tissue. This means that terms of these entity types are frequently made up of common words. The current system performs no break-up of term as a split-up does not improve the accuracy of annotation.
- The **stop-words** feature computes whether the n-gram is a stop-word. Some stop-words also have a biomedical meaning and therefore appear in terminology resources. The NN can give lower weights to these words to decrease the rate of false-positives produced by these words. We used NLTKs [57] English stop-word list, which comprises 153 words.
- The **word embeddings** feature fetches the word embedding of an n-gram. Word embeddings add distributional knowledge for a given word. In our model, we used the pre-trained embeddings of [58], which target biomedical applications. For multi-word terms, which have no embedding, we used to take the word embedding of the head token, using the last token as an approximation which typically conveys the main meaning. The current system, however, performs an individual look-up for every token in the term and averages their respective embeddings using the mean to produce a single vector. We found that this improved the F1-scores for NER and CR by 0.3–0.4%, compared to using the word embedding of the head token.

Experiments have shown that word embeddings are the most salient feature. In fact, using only word embeddings and excluding all other features only produced a small drop of 1 to 2% in the F1-score on the CRAFT corpus. This suggests that the influence of the other features is not very pronounced and that they might be redundant in future work. The public OGER web service uses three features only (common dictionary, stop-words, word embeddings).

**Fig. 4:**
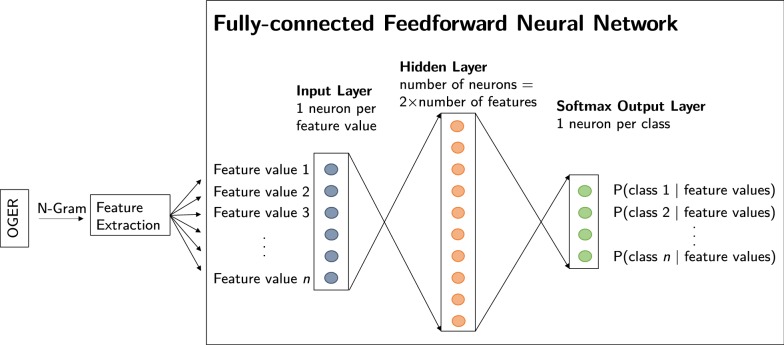
Architecture of the NN

A third main difference is that the previous system [32] trained separate NNs for each entity type, where a single output neuron makes a basic accept/reject decision given some threshold value. Our new system, however, trains a joint model by constructing a softmax output layer that computes a probability distribution over all entity types, as shown in Fig. 4. This has the advantage that the probabilities of different entity types become comparable and that only one model has to be loaded for predictions.

To give the NN filter capabilities, an additional output neuron for the label “not an entity” was added. For training, we used the rest of the words from the CRAFT corpus that were not explicitly annotated as biomedical in order for the NN to learn how common words look like. Note that the NN only receives single words as input in the case of common words, while in the case of biomedical entities, it can receive multi-word examples. The downside of this strategy is that the NN does not learn to remove irrelevant multi-word matches produced by the up-stream annotator.

To allow for multiple classifications of the same n-gram, as is the case for some biomedical datasets (e.g. the CRAFT corpus), entity types with the second-highest probability are also considered by defining a maximum probability difference to the most probable entity type.

### Server architecture

**Fig. 5:**
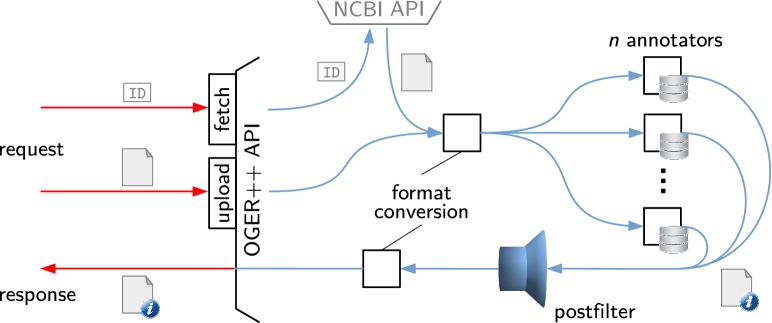
System architecture of the OGER++ server

An overview of the server architecture is given in Fig. 5. Incoming requests are expected to either include a PubMed or PMC ID (fetch command), or to contain an entire document in the request payload (upload command). In the case of a fetch request, the service fetches the referenced document using NCBI’s efetch API [59]. The client can specify a number of parameters through the URL and an optional query string, such as the document input and output formats or the selection of terminologies to use for annotation. Different terminologies are maintained in separate instances of the dictionary-based annotation component as described above, called annotators. New annotators can be created by the client through another request (dict command, not shown in the figure); the Bio Term Hub makes use of this features to allow users to send newly compiled terminology resources to OGER. After annotation, the documents are passed to the postfilter for disambiguation and serialized into the requested output format, before being returned to the client.

## Results and discussion

We assessed OGER++ with benchmarks for processing speed, an analysis of entity-type ambiguity, and an evaluation of annotation quality, as is discussed in the following sections.

### Processing speed

The *technical interoperability and performance of annotation servers* (TIPS) task of the BioCreative V.5 challenge was a shared task designed to evaluate the efficiency and reliability of annotation servers in the biomedical domain. Among the participating systems, OGER was the fastest system (best results for *average response time* and *mean time per document volume*, team 122 in [60]). Additionally, we recently performed a series of benchmarks for measuring the processing speed of OGER++. The results are summarized in Table 1. We analyzed two different document sizes (abstracts vs. full-text) and two different input formats (plain-text vs. NCBI XML). The same random sample of PubMed abstracts and PMC full-text documents was used for the different input formats.

**Table 1:** Average processing time analysis for different document formats and sizes

Size	Format	Documents	doc/s	kiB/s	ann/s	kiB/s (macro)	ann/s (macro)	ann/doc
Abstracts	txt	1000	9.73	8.27	462.96	11.75	239.70	47.56
Abstracts	xml	1000	9.45	57.26	449.43	222.44	241.55	47.56
Full-text	txt	529	0.89	16.97	866.89	18.95	621.95	979.09
Full-text	xml	529	0.88	47.44	862.01	64.16	620.00	979.37
Full-text (no disambiguation)	txt	529	17.82	341.64	28072.22	350.24	18569.08	1575.27

For kiB/s and ann/s, micro- and macro-average are given separately

The benchmarks were carried out using the public OGER web API. This web service is hosted on a virtual machine with 16 shared CPU cores and 128 G exclusive RAM. Each document was processed with a separate HTTP request in a serial fashion (no parallelization). Due to the requests being sent from the same physical machine on which the OGER service is run, network latency is expected to have negligible effect on the measurements; therefore, these results are not comparable to the average response time measured in the TIPS task (1.1 s per abstract, i.e. 10 times slower), where three separate HTTP requests between distant servers were necessary for each document. However, the current figures include the overhead required by the HTTP protocol. During the time of the tests, the server did not have a heavy load; in busy times, the processing times can be up to three times higher, even though OGER’s service machine is prioritized by default.

Most time is spent in disambiguation, i.e. the NN predicting probabilities for each annotation. This can be clearly seen by comparing to the last line in the table, where full-text documents were processed without disambiguation, which leads to 20 times faster processing on average. Document size affects processing time greatly, as abstracts are processed more than 10 times faster than full-text documents. This is best explained by the higher number of annotated terms in longer texts. The input format has only a marginal effect both on processing time and the number of annotations the absence of structural mark-up tends to accelerate processing and has an influence on term matching.

### Entity-type ambiguity

**Fig. 6:**
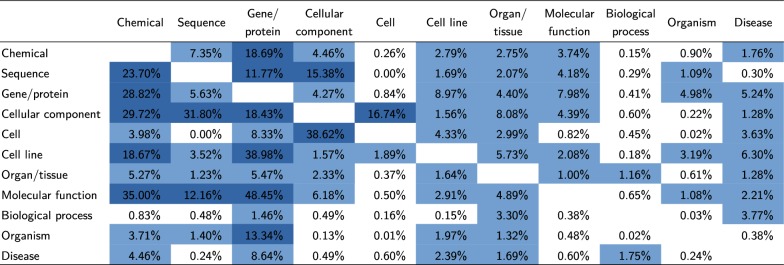
Name overlap among different entity types. The figures in each row denote the percentage of names with this type that are also annotated with the type of the respective column. For example, of all mentions annotated as cell line, close to 39% also have a gene/protein annotation, while only 9% of the gene-annotated mentions also have an annotation as cell line

In order to estimate the degree of ambiguity in a multi-type entity-recognition setting, we performed an experiment using OGER without its disambiguation module. Using a large dictionary with 5.6 million names for a total 2.9 million concepts of 11 different entity types, we automatically annotated a random sample of 3 million PubMed abstracts. Since disambiguation was disabled, each annotated mention was tagged with one or more entity types. We used these data to compute a confusion matrix of names that are shared among different entity types, measured by their occurrence in the scientific literature. When comparing dictionary entries in their exact spelling, there is almost no overlap across entity types; however, the relaxed matching scheme used for annotation introduces a significant number of collisions, as can be seen in Fig. 6. Please note that the true type is unknown in this setting, and that a considerable fraction of annotations is potentially spurious, i.e. words of common language that are erroneously annotated as a biomedical entity. However, these figures give a realistic estimate of how hard the task of the disambiguation module is.

### CRAFT evaluation

We performed an evaluation on 20 articles from the CRAFT corpus using the metrics precision, recall and F1-score. We evaluated the correctness of the system output at two different levels: entity type (NER evaluation) and identifier (CR evaluation), as is described in the following sections.

#### NER evaluation

In the NER-level evaluation, we considered a prediction to be correct (true positive) if it matched the span (character offsets) and entity type of a ground-truth annotation. We required the span to match exactly, i.e. no credit was given for predictions that partially overlapped with a true annotation. Table 2 shows micro-averaged precision, recall and F1-scores broken down by entity type for three different systems: the knowledge-based system (OG), the previous hybrid system (OG + Dist) and the new hybrid system (OG + Joint). Using the new NN architecture along with the new features yielded a 1% increase in the overall F1-score compared to the former hybrid system. Looking at specific entity types, the new hybrid system outperforms the other two systems in four out of the seven entity types. The new hybrid system achieves better F1-scores due to more balanced precision (65%) and recall scores (79%), while the former hybrid system has high precision (88%), but a lower recall (58%).

**Table 2:** Evaluation at the level of NER

Entity type	Precision			Recall			F1		
	OG	OG + Dist	OG + Joint	OG	OG + Dist	OG + Joint	OG	OG + Dist	OG + Joint
All	0.44	0.88	0.800	0.62	0.58	0.645	0.51	0.70	0.714
Chemicals	0.44	0.89	0.870	0.73	0.68	0.726	0.55	0.77	0.792
Cells	0.88	0.88	0.738	0.77	0.67	0.748	0.80	0.76	0.743
BPMFs	0.39	0.78	0.628	0.25	0.22	0.349	0.30	0.35	0.449
Cellular components	0.51	0.91	0.867	0.60	0.56	0.658	0.55	0.70	0.748
Organisms	0.29	0.98	0.977	0.92	0.91	0.920	0.44	0.94	0.948
Proteins	0.49	0.86	0.778	0.84	0.75	0.812	0.62	0.80	0.795
Sequences	0.46	0.89	0.833	0.67	0.64	0.670	0.54	0.75	0.743

#### CR evaluation

In the evaluation at the level of Concept Recognition, a prediction was seen as correct if a ground-truth annotation existed at the same position with the same concept identifier. Again, we required the spans to be identical. Table 3 shows the performance of the knowledge-based system (OG), the previous hybrid system (OG + Dist) and the new hybrid system (OG + Joint) with respect to micro-averaged precision, recall and F1-scores in a strict evaluation scheme (no credit for partially overlapping spans). The overall F1-score of the new hybrid system (OG + Joint) improved by 7% compared to the previous hybrid system (OG + Dist). The difference is even more pronounced for the knowledge-based system (+ 27%). The higher F1-score increased mostly due to a much better overall precision (+ 14%), while the overall recall score only improved by 1%. In total, the new hybrid system outperforms the previous one in three and ties with four out of the seven entity types in terms of F1-scores.

**Table 3:** Evaluation at the level of concept recognition

Entity type	Precision			Recall			F1		
	OG	OG + Dist	OG + Joint	OG	OG + Dist	OG + Joint	OG	OG + Dist	OG $+$ Joint
All	0.32	0.51	0.650	0.52	0.49	0.503	0.40	0.50	0.567
Chemicals	0.28	0.59	0.601	0.61	0.57	0.568	0.39	0.58	0.584
Cells	0.88	0.87	0.878	0.72	0.66	0.713	0.79	0.75	0.787
BPMFs	0.35	0.72	0.634	0.19	0.17	0.178	0.25	0.27	0.278
Cellular components	0.49	0.87	0.930	0.59	0.56	0.581	0.54	0.68	0.716
Organisms	0.16	0.49	0.486	0.71	0.70	0.709	0.26	0.58	0.577
Proteins	0.45	0.84	0.788	0.83	0.74	0.799	0.59	0.79	0.794
Sequences	0.27	0.59	0.561	0.53	0.51	0.516	0.36	0.54	0.537

#### Error analysis

Most false positives (FPs) are introduced by the aggressive matching algorithm of OGER. For example, the match ‘IOP) [1’ is returned for the string ‘elevated intraocular pressure (IOP) [1–5]’, as its collapsed form ‘IOP1’ is present in the terminologies. Another example is ‘at 1’, which is extracted from the string ‘at 1 minute’ because the term ‘AT-1’ has the normalized form ‘at 1’. The postfilter fails to remove these two cases because the NN is largely trained on single words as input and only receives multi-word terms if it denotes a ground-truth entity. Thus, it never observes multi-word examples that are labeled as non-biomedical and learns that multi-word terms are always relevant. Another source of error are terms that are located within a word. For instance, the word ‘Thr164Ala’ contains the terms ‘Thr’ and ‘Ala-’ (normalized as ‘Ala’). Some FPs are also common words such as ‘processes’ and ‘positions’ that also occur in terminologies and a small number are wrong re-classifications of the same span by the postfilter.

Most false negatives (FNs) are also caused by the knowledge-based system. While the postfilter can remove all types of FPs, it can only rectify FNs with the same span through re-classification, but not FNs with diverging spans, as these are pre-determined by the knowledge-based system. The vast majority of FNs are terms that are not listed verbatim in the terminologies:

- Morphological variations of the terms, e.g. ‘carbonic’ ($\rightarrow$ ‘carbon’), ‘mammalian’ ($\rightarrow$ ‘Mammalia’)
- Abbreviations, e.g. ‘bp’ ($\rightarrow$ ‘base pair’), ‘Chr’ ($\rightarrow$ ‘chromosome’)
- Synonyms, e.g. ‘blood flow’ ($\rightarrow$ ‘blood circulation’), ‘chow’ ($\rightarrow$ ‘food’)
- Ellipses, e.g. ‘A to G’ ($\rightarrow$ ‘A to G transition’), ‘alteration’ ($\rightarrow$ ‘sequence alteration’)
- Hyponyms, e.g. ‘depression’ ($\rightarrow$ ‘negative regulation of biological process’), ‘passes’ ($\rightarrow$ ‘establishment of localization’).

Terms linked via the hyponym-hyperonym relation make up the largest group of these FNs and are pervasive for biological processes and molecular functions, whose recall is accordingly very low.

## Conclusions

We have presented a fast, efficient, reliable entity NER-CR system for biomedical scientific literature. Competitive performance has been demonstrated by participation in a shared task and separate evaluations presented in this paper.

Besides fixing some of the remaining problems revealed by the error analysis presented in this paper, we are also currently extending our experiments to multiple corpora, with different annotation strategies, with the goal of achieving competitive performance on several of them using a common architecture. We are also experimenting with more complex neural networks for the filtering stage, in particular recurrent NNs.

## Abbreviations

API: application programming interface
BPMF: biological processes and molecular functions
ChEBI: chemical entities of biological interest
CR: concept recognition
CRAFT: Colorado Richly Annotated Full Text
CRF: conditional random fields
CTD: Comparative Toxicogenomics Database
FN: false negative
FP: false positive
HTTP: Hypertext Transfer Protocol
JSON: JavaScript Object Notation
MeSH: Medical Subject Headings
NCBI: US National Center for Biotechnology Information
NER: named entity recognition
NLP: natural language processing
NLTK: Natural Language Toolkit
NN: neural network
OGER: OntoGenes entity recognizer
PMC: PubMed Central
REST: Representational State Transfer
TIPS: Technical interoperability and performance of annotation servers
URL: Unified Resource Locator
XML: Extensible Markup Language
